# 2-Hydroxychalcone as a Potent Compound and Photosensitizer Against Dermatophyte Biofilms

**DOI:** 10.3389/fcimb.2021.679470

**Published:** 2021-05-13

**Authors:** Níura Madalena Bila, Caroline Barcelos Costa-Orlandi, Carolina Orlando Vaso, Jean Lucas Carvalho Bonatti, Letícia Ribeiro de Assis, Luís Octavio Regasini, Carla Raquel Fontana, Ana Marisa Fusco-Almeida, Maria José Soares Mendes-Giannini

**Affiliations:** ^1^ Department of Clinical Analysis, School of Pharmaceutical Sciences, Universidade Estadual Paulista (UNESP), Araraquara, Brazil; ^2^ Department of Para-Clinic, School of Veterinary, Universidade Eduardo Mondlane (UEM), Maputo, Mozambique; ^3^ Department of Chemistry and Environmental Sciences, Institute of Biosciences, Humanities and Exact Sciences, Universidade Estadual Paulista (UNESP), Sao Jose do Rio Preto, Brazil

**Keywords:** dermatophytes, biofilms, 2-chalcone, photodynamic therapy, mechanism of action, *Trichophyton rubrum*, *T. mentagrophytes*

## Abstract

Dermatophytes, fungi that cause dermatophytosis, can invade keratinized tissues in humans and animals. The biofilm-forming ability of these fungi was described recently, and it may be correlated with the long treatment period and common recurrences of this mycosis. In this study, we evaluated the anti-dermatophytic and anti-biofilm activity of 2-hydroxychalcone (2-chalcone) in the dark and photodynamic therapy (PDT)-mediated and to determine its mechanism of action. *Trichophyton rubrum* and *Trichophyton mentagrophytes* strains were used in the study. The antifungal susceptibility test of planktonic cells, early-stage biofilms, and mature biofilms were performed using colorimetric methods. Topographies were visualized by scanning electron microscopy (SEM). Human skin keratinocyte (HaCat) monolayers were also used in the cytotoxicity assays. The mechanisms of action of 2-chalcone in the dark and under photoexcitation were investigated using confocal microscopy and the quantification of ergosterol, reactive oxygen species (ROS), and death induction by apoptosis/necrosis. All strains, in the planktonic form, were inhibited after treatment with 2-chalcone (minimum inhibitory concentration (MIC) = 7.8-15.6 mg/L), terbinafine (TRB) (MIC = 0.008–0.03 mg/L), and fluconazole (FLZ) (1–512 mg/L). Early-stage biofilm and mature biofilms were inhibited by 2-chalcone at concentrations of 15.6 mg/L and 31.2 mg/L in all tested strains. However, mature biofilms were resistant to all the antifungal drugs tested. When planktonic cells and biofilms (early-stage and mature) were treated with 2-chalcone-mediated PDT, the inhibitory concentrations were reduced by four times (2–7.8 mg/L). SEM images of biofilms treated with 2-chalcone showed cell wall collapse, resulting from a probable extravasation of cytoplasmic content. The toxicity of 2-chalcone in HaCat cells showed higher IC_50_ values in the dark than under photoexcitation. Further, 2-chalcone targets ergosterol in the cell and promotes the generation of ROS, resulting in cell death by apoptosis and necrosis. Overall, 2-chalcone-mediated PDT is a promising and safe drug candidate against dermatophytes, particularly in anti-biofilm treatment.

## Introduction

Dermatophytes are filamentous fungi that may infect keratinized structures such as the skin, hair, and nails of humans and animals, causing dermatophytosis (Costa-Orlandi et al., 2014; Heidrich et al., 2015; Maraki and Mavromanolaki, 2016). This disease is globally considered as the most common dermatological zoonosis, with a prevalence of 20–25% in the global human population (Ivaskiene et al., 2016).

Phylogenetic re-taxonomy has been proposed for dermatophytes, suggesting their division into six genera (Zhan and Liu, 2017) and into nine genera (De Hoog et al., 2017). Nevertheless, the principal genera include *Trichophyton, Microsporum*, and *Epidermophyton* (Moriello, 2004; Costa-Orlandi et al., 2014; Maraki and Mavromanolaki, 2016; De Hoog et al., 2017). Dermatophytes can be grouped as anthropophilic, zoophilic, and geophilic (Moriello, 2004; Ivaskiene et al., 2016). *T. rubrum* is the most prevalent dermatophyte species, accounting for more than 80% of all infections. This species is commonly isolated in cases of *tinea unguium*, *tinea corporis*, *tinea cruris*, and *tinea pedis* (Faway et al., 2016; Zhan and Liu, 2017).

One of the main virulence factors of fungal species is the formation of communities called biofilms (Costa-Orlandi et al., 2017). *In vitro* biofilm formation by two of the most prevalent dermatophyte species, *T. rubrum* and *T. mentagrophytes*, was described (Costa-Orlandi et al., 2014) and was soon demonstrated in *Microsporum canis* (Danielli et al., 2017). *In vivo* biofilm formation by dermatophytes in patients with dermatophytosis is currently under investigation. However, a hypothetical relationship has been reported between biofilm formation and the persistent clinical condition of onychomycosis infections that have firm white masses of adhesion and are challenging to remove and treat (Burkhart; Burkhart; Gupta, 2002; Gupta, Daigle,Carviel, 2016; Gupta and Foley, 2019).

Although a reasonable number of antifungal drugs are available for treating dermatophytosis, there has been little progress in new drug development in the last two decades, and recurrences in onychomycosis cases have increased by 50% (Gupta, Daigle, Carviel, 2016; Singh et al., 2018a). Most of these drugs are classified into two families: azoles and allylamines (Gupta and Cooper, 2008; Aggarwal and Goindi, 2012). Azole derivatives, other than terbinafine and naftifine, are commonly prescribed to topically treat superficial infections in the early stages (Makimura et al., 1999; Gupta and Cooper, 2008; Aggarwal and Goindi, 2012). In extensive infections, chronic infections, or onychomycosis, oral drugs such as terbinafine, itraconazole, fluconazole, griseofulvin, and ketoconazole are used (Gupta and Cooper, 2008; Aggarwal and Goindi, 2012; Aggarwal et al., 2020). Due to the long treatment period for these infections, coupled with the considerable toxicity of most oral antifungal agents, monitoring is necessary for the hepatic, renal, and hematopoietic functions (Gupta and Cooper, 2008; Gupta,Versteeg, Shear, 2017a).

Difficult eradication, drug toxicity, and high recurrence rate have encouraged the search for therapeutic alternatives for dermatophytosis, mainly if they are associated with biofilm formation. The anti-dermatophyte effects of essential oils from plants (Mahboubi; Heidarytabar; Mahdizadeh, 2017), plant extracts (Mahboubi; Kazempour, 2015; Sun et al., 2017), ozone gas, ozonized oil (Ouf et al., 2016), and photodynamic inactivation (Paz-Cristobal et al., 2014; De Oliveira et al., 2015; Shamali et al., 2018), have already been described.

Chalcones or 1,3-diphenyl-2-propen-1-one are polyhydroxy compound precursors for flavonoids and isoflavonoids, which are abundant in fruits, vegetables, and edible plants (Gupta; Jain, 2015; Fu et al., 2016; Karimi-Sales; Mohaddes; Alipour, 2017). However, these compounds can be synthesized in the laboratory using the Claisen–Schmidt condensation method (Karimi-Sales; Mohaddes; Alipour, 2017). Chalcones demonstrate many biological activities such as anti-inflammatory, anti-tumor (Khanapure et al., 2018), antioxidant (Singh et al., 2018b), antidiabetic (Cai et al., 2017), antibacterial (Singh et al., 2018a), antifungal (Gupta; Jain, 2015; Fu et al., 2016; Illicachi et al., 2017), antiprotozoal (Illicachi et al., 2017; Tajuddeen et al., 2018), and antiparasitic (Kotlyar et al., 2019), as well as activities against Alzheimer’s disease (Zhang et al., 2018b) and cholinesterase inhibition (Shah et al., 2018) among others. Chalcones can be excellent photosensitizers for PDT. For instance, photodynamic inactivation has been demonstrated in *Histoplasma capsulatum* (Melo et al., 2017).

PDT is a non-invasive treatment comprising the use of laser light or light-emitting diode **(**LED) of a specific wavelength that activates a photosensitive agent in the presence of oxygen, which results in the production of reactive oxygen species (ROS), free radicals, and consequently, cell death (Davies et al., 2016; De Figueiredo Freitas et al., 2017; Yang et al., 2018; Yuan et al., 2017). PDT is a promising modality as it is effective against a wide range of microorganism species (De Figueiredo Freitas et al., 2017). Photodynamic inactivation of microorganisms can occur in strains that are susceptible or resistant to conventional drugs (Mai et al., 2017). Therefore, this study aimed to evaluate the efficacy of 2-chalcone in the dark and of 2-chalcone-mediated PDT against dermatophyte biofilms, as well as to investigate its toxicity and mechanism of action in human skin keratinocytes (HaCat).

## Materials And Methods

### Microorganisms and Synthesis of 2-Chalcone

*T. rubrum* ATCC 28189, ATCC MYA-4438, and *T. mentagrophytes* ATCC 11481 were used in this study. All strains were obtained from the Clinical Mycology Laboratory of the Department of Clinical Analysis, Faculty of Pharmaceutical Sciences-UNESP, Brazil. Microorganisms were grown on malt extract agar [malt extract (Kasvi): 2%, peptone from animal tissue (*Sigma-Aldrich*): 2%, glucose (*Synth*): 2% and agar (Kasvi): 2%], pH 5.7, incubated at 28°C for 7 days or until sporulation (Garcia et al., 2020).

Synthesis of 2-chalcone was performed as described by Melo et al. (2017) in collaboration with Prof. Dr. Luis Octávio Regasini’s research group from the Institute of Biosciences, Letters and Exact Sciences-UNESP, Brazil. The compound was functionalized by adding one hydroxyl group on carbon-2 to increase its solubility in aqueous medium.

### *In Vitro* Susceptibility of Dermatophytes to 2-Chalcone and Antifungal Drugs and Determination of Minimum Fungicide Concentration (MFC)

#### Determination of the Minimum Inhibitory Concentration (MIC)

Susceptibility tests were performed according to the document M38-A2, proposed by the Clinical Laboratory Standards Institute (CLSI) (2008), with a minor modification of addition of resazurin (Costa-Orlandi et al., 2020). Briefly, 2-chalcone was solubilized in 100% dimethyl sulfoxide (DMSO) at a stock concentration of 30,000 mg/L and was stored at −80°C. The working solutions of 2-chalcone at concentrations ranging from 0.12 to 62.5 mg/L were prepared in Roswell Park Memorial Institute (RPMI)-1640 medium with L-glutamine, without sodium bicarbonate, and with phenol red as the pH indicator (*Gibco^®^*), and buffered with 4-Morpholinepropanesulfonic acid hemisodium salt (MOPS) (*Sigma-Aldrich*), pH = 7. TRB (*Sigma-Aldrich*) (0.5–0.001 mg/L), FLZ (*Sigma-Aldrich*) (0.12–64 mg/L), and the *T. rubrum* ATCC MYA-4438 were also used for quality control. Fungal suspensions were prepared in 0.85% NaCl and conidia were adjusted to a final concentration of 2.5 × 10^3^ cells/mL using a hemocytometer, before adding to microdilution plates. Dilutions of the compound and antifungal drugs were dispensed in a 96-well microplate (Kasvi) at a total volume of 100 μL/well, following 100 µL of the inoculum. Visual and colorimetric readings were performed by adding 30 µL of 0.03% resazurin. Resazurin is a blue reagent that is reduced to pink-colored resorufin in the presence of viable cells (Scorzoni et al., 2007; Costa-Orlandi et al., 2014; Aneke; Otranto; Cafarchia, 2018).

#### Determination of the MFC

The MFC was determined as described by Costa-Orlandi et al. (2020). Aliquots from each well of the MIC assay microdilution plate were added to Petri dishes containing Sabouraud Dextrose agar (BD Difco™) and incubated at 28°C for 96 hours. The MFC is defined as the lowest concentration of the compound that shows no growth of fungal colonies.

### Susceptibility Assay of Early-Stage Biofilms and Mature Biofilms Against 2-Chalcone and Antifungal Drugs

The biofilm susceptibility assay was based on the protocol described by Pierce et al. (2008) modified by Costa-Orlandi et al. (2020). Early-stage (24 hours) and mature (96 hours) biofilms, formed in the 96-well plates as described by Costa-Orlandi (2014), were washed with sterile phosphate-buffered saline (PBS). Two hundred microliters of 2-chalcone solution and antifungals diluted in RPMI-1640 medium were added to the plates containing biofilms and incubated at 37°C for 96 hours. Metabolic activity was then measured using the XTT (2,3-Bis-(2-Methoxy-4-Nitro-5-Sulfophenyl)-2H-Tetrazolium-5-Carboxanilide) reduction assay. Inhibition was considered as the reduction of at least 50% metabolic activity compared to the control without treatment.

### Effect of Antifungal Drugs and 2-Chalcone on Planktonic Cells (10^6^ cells/mL)

The protocol used to evaluate antifungal drugs and 2-chalcone in biofilms was also applied to the planktonic cells and at the same concentration used for biofilm formation (1 × 10^6^ cells/mL) to compare the susceptibility of cells in the two different forms. Fungal suspensions were prepared to obtain a final concentration of 1 × 10^6^ cells/mL. One hundred microliters of suspensions placed in the 96-well plates (*Kasvi*), 100 µL of the working solutions of the compound (1–500 mg/L) and the antifungal agents TRB (0.002–1 mg/L) and FLZ (1–512 mg/L) were diluted in RPMI-1640 medium with the same specifications previously described. The plates were then incubated at 37°C for 96 hours, and the XTT reduction assay was used to quantify the metabolic activity of cells as described by Martinez and Casadevall (2006) and Pierce et al. (2008) with minor modifications. Briefly, 50 µL of the XTT solution and 4 µL of menadione were added to the suspensions in the wells and incubated for 3 hours. The absorbance was measured using a microplate reader (Epoch, Biotek) at 490 nm

### Photodynamic Therapy Assay

A blue LED (IrradLED^®^-Biopdi, Sao Carlos, SP, Brazil) with a wavelength range between 455 nm and 492 nm, was used as the light source. Intensity was maintained at 58 mW/cm^2^, and the administered dose was 150 J/cm^2^. The photosensitization assay was performed as described by Baltazar et al. (2013), with minor modifications. Briefly, biofilms were prepared as previously described, without phenol red in RPMI-1640 (*Sigma-Aldrich)* medium. Then, early-stage and mature biofilms were washed with sterile PBS and placed in contact with different concentrations of 2-chalcone (0.25–125 mg/L). The plates were incubated for 10 minutes in the dark and at 25°C and were then irradiated. After irradiation, the plates were incubated at 37°C for 96 hours. The analysis of the metabolic activity of cells was done by the XTT reduction assay.

### Topographic Analysis of Biofilms by Scanning Electron Microscopy (SEM)

Early-stage and mature biofilms treated with 2-chalcone were processed as described by Martinez et al. (2010) and Costa-Orlandi et al. (2014). Biofilms were formed in 24-well plates, washed three times with PBS, and fixed with 800 µL of 2.5% glutaraldehyde solution (*Sigma-Aldrich*) for 1 hour at 4°C. The samples were then dehydrated with increasing ethyl alcohol concentrations (50–100%) at 25°C and were subsequently dried under the same conditions. Before microscopic analysis, the plate’s bottom containing the samples was cut with a scalpel, mounted on aluminum cylinders with silver (stubs), and placed on a high vacuum evaporator (Denton Vacuum Desk V, Jeol USA) for gold plating. The damage to the biofilm topography was analyzed using a scanning electron microscope Jeol JSM-6610LV at the School of Dentistry, UNESP-Araraquara, Brazil.

### Cytotoxicity Assay for 2-Chalcone in Human Skin Keratinocytes (HaCat)

The cytotoxicity assay aimed to verify the selectivity index (SI) after the treatment of planktonic cells and mature biofilms with 2-chalcone in the dark and 2-chalcone-mediated PDT. The assay was performed as described by Costa-Orlandi et al. (2020) with minor modifications. Briefly, HaCat cells (CLS Cell Lines Service, 300493) were maintained in cell culture bottles with Dulbecco′s modified eagle′s medium (DMEM), containing 10% fetal bovine serum without phenol red, and incubated under standard conditions (37°C, 5% CO_2_). Cell suspensions were prepared to obtain a final concentration of 2 × 10^4^ cells/well in a 96-well microdilution plate. After 24 hours of incubation, the culture medium was removed and 200 μL of different concentrations of 2-chalcone were added. On some plates, photosensitization of 2-chalcone at 150 J.cm^-2^ was performed, and the others were kept in the dark. All the plates were incubated for 72 hours in the same conditions and protected from light. After incubation, 20 μL of resazurin (*Sigma-Aldrich*) at 60 μM was added and the plates were further incubated for 8 hours. Cell viability was assessed based on spectrophotometric (Epoch, Biotek) analysis at wavelengths 570 nm and 600 nm.

### Determination of the Mechanism of Action

#### Laser Scanning Confocal Microscopy

Confocal microscopy was used to evaluate the damage to the cell wall caused by 2-chalcone, using fluorochrome Calcofluor White (*Thermo Fisher Scientific*) and *T. rubrum* ATCC 28189. Fungal suspensions were prepared at a concentration of 1 × 10^6^ cells/mL and added to 24-well plates containing sterile coverslips, along with sub-inhibitory doses of 2-chalcone (7.8 mg/L). After the incubation period (35°C, for 96 hours), the supernatant was removed and the coverslips were washed with PBS. The coverslips were covered with Calcofluor White solution (100 mg/L), and the plates were incubated again at 37°C, for 45 minutes, protected from light. Then, the coverslips were washed with PBS, removed from the wells, and mounted on 4 μL of Fluoromount-G (*Sigma-Aldrich*), which was previously deposited on microscopic slides. The slides were then observed using a confocal microscope (Carl Zeiss LSM 800 with Airyscan) with an image capture and processing program (Software ZEN BLUE 2.3 System) at the Faculty of Dentistry, UNESP-Araraquara, Brazil (Curcio et al., 2017; Oliveira et al., 2020).

#### Quantification of Membrane Ergosterol

Ergosterol quantification was performed as described by Arthington-Skaggs et al. (1999) with minor modifications. Briefly, sub-inhibitory concentrations of 2-chalcone in the dark (7.8 mg/L), 2-chalcone-mediated PDT (1 mg/L), FLZ (128 mg/L), and amphotericin B (AMB) (1 mg/L) were used to treat a suspension of *T. rubrum* ATCC 28189 at a final concentration of 1 × 10^6^ cells/mL (diluted in RPMI-1640). The samples were incubated at 35°C, under agitation at 150 rpm for five days. After incubation, the samples were centrifuged and washed with sterile distilled water, and 3 ml of 25% alcoholic KOH solution was added. For sterol extraction, the samples were incubated in a water bath at 85°C for 1 hour after being transferred to glass tubes with a screw cap. The samples were cooled at 25°C, mixed with 1 mL of sterile distilled water, 3 mL of n-heptane, and sterile glass beads, followed by homogenization for 10 minutes on a vortex. The resulting supernatant (n-heptane layer) was transferred to microtubes and incubated at −20°C for 24 hours followed by an analysis on a visible UV spectrophotometer at a wavelength of 281 nm. Standard curves were prepared using 95% pure ergosterol at concentrations ranging from 75 to 10 mg/L.

#### Apoptosis/Necrosis Assay

The death mechanism in *T. rubrum* ATCC 28189 was studied after treatment with 2-chalcone in the dark, 2-chalcone-mediated PDT, AMB, and FLZ. Inocula were prepared and adjusted to a final concentration of 1 × 10^6^ cells/mL in a volume of 1.5 mL. The cells were treated with the same volume of compounds and AMB at a concentration of 4x MIC. FLZ treatment was performed at a concentration of 256 mg/L (as this strain was found to be resistant to FLZ). Cell death was evaluated using the Annexin V-FITC apoptosis detection kit (*Sigma-Aldrich*, A9210) following the manufacturer’s guidelines. The samples were analyzed on a BD FACS Canto I flow cytometer located at the Clinical Mycology Laboratory at the School of Pharmaceutical Sciences, UNESP-Araraquara, Brazil.

#### Quantification of ROS

Intracellular ROS production after treating *T. rubrum* ATCC 28189 with 2-chalcone in the dark and 2-chalcone mediated PDT was evaluated using 50 µM H2DCFDA (2.7 dichlorodihydrofluorescein diacetate, *Invitrogen*) as described by Singulani et al. (2019). This compound is converted to a highly fluorescent 2ʹ, 7ʹ-dichlorofluorescein (DCF) compound after cleavage of its acetate group by intracellular esterases and this compound binds to ROS. As controls, AMB and FLZ treatments were used. The treatment was performed as described apoptosis/necrosis assay section. After the incubation period, the samples were washed, following suspension in 500 µL of PBS, and transferred to cytometer tubes. Then, 1.5 µL of the H2DCFDA solution was added with subsequent incubation at 25°C in the dark for 10 minutes. The samples were analyzed on a BD FACS Canto I flow cytometer.

### Data Analysis

All data from this study are representative of at least three independent and triplicate experiments. GraphPad Prism 5.0 software (GraphPad Software Inc., La Jolla, CA) was used to construct graphs and for statistical analysis. Non-linear semi-log regression was performed to obtain the IC_50_ for treated HaCat cells. Analysis of variance with Bonferroni post-hoc test was applied to the other graphs. Differences with p < 0.05 were considered statistically significant.

## Results

### Determination of MIC and MFC

The results of the antifungal activities of 2-chalcone, TRB, and FLZ against dermatophyte species are shown in **Table 1**. In all strains tested, 2-chalcone had a MIC of 7.8 mg/L. TRB had a MIC of 0.03 mg/L in *T. rubrum* strains and 0.008 mg/L in *T. mentagrophytes* strain. On the contrary, *T. rubrum* ATCC 28189 was resistant to FLZ with a MIC of 64 mg/L, whereas the remaining strains, *T. rubrum* ATCC MYA-4438 and *T. mentagrophytes* ATCC 11481, were susceptible to FLZ (with MIC 4 mg/L and 1 mg/L, respectively).

**Table 1:** Antifungal activity (expressed in mg/L) of 2-chalcone, terbinafine, and fluconazole against dermatophyte species.

	*T. rubrum* ATCC 28189		*T. rubrum* ATCC MYA-4438		*T. mentagrophytes* ATCC 11481	
	MIC	MFC	MIC	MFC	MIC	MFC
**2-Chalcone**	7.8	15.6	7.8	15.6	7.8	15.6
**TRB**	0.03	0.06	0.03	0.06	0.008	0.016
**FCZ**	64	>64	4	32	1	16

TRB, terbinafine; FCZ, fluconazole; MIC, minimum inhibitory concentration; MFC, minimum fungicidal concentration.

The MFC for 2-chalcone corresponded to 15.6 mg/L, double the MIC, in all strains tested. The same trend was observed in the TRB results. FLZ had a MFC of 16, 32, and > 64 mg/L in *T. mentagrophytes* ATCC 11481, *T. rubrum* ATCC MYA-4438, and *T. rubrum* ATCC 28189, respectively.

### Effect of 2-Chalcone and Antifungal Drugs on Early-Stage and Mature Biofilms

**Figure 1:**
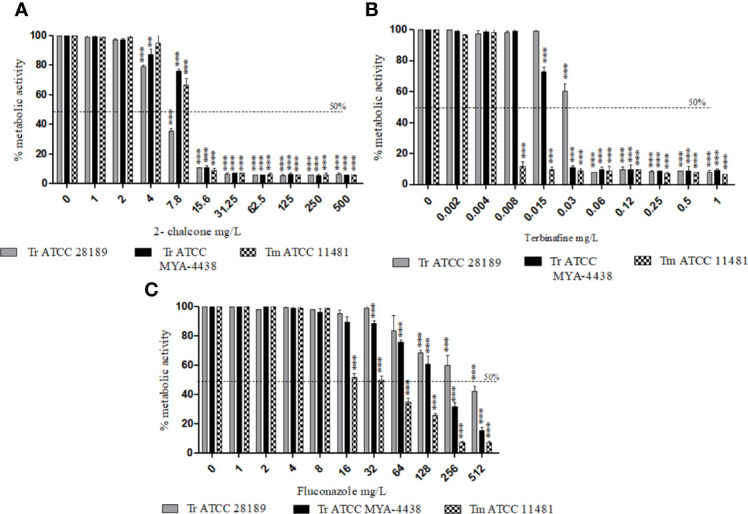
Effect of 2-chalcone **(A)**, terbinafine **(B)**, and fluconazole **(C)** on early-stage biofilms of *T. rubrum* ATCC 28189, *T. rubrum* ATCC MYA-4438, and *T. mentagrophytes* ATCC 11481 measured using the XTT reduction assay. The compounds 2-chalcone and terbinafine inhibited the metabolic activity of early-stage biofilms from the concentrations of 15.6 and 32 mg/L in all strains tested. Biofilms formed by both the *T. rubrum* strains conferred resistance to fluconazole at all concentrations tested. Biofilms of the *T. mentagrophytes* strain were inhibited from the concentration of 64 mg/L (**p < 0.01; ***p < 0.001). Tr, *T. rubrum*; Tm, *T. mentagrophytes*.

From 15.6 mg/L, 2-chalcone inhibited the metabolic activity of early-stage biofilms of the three strains tested, with approximately 90% reduction in cell viability when compared to the control without treatment (p < 0.001) (**Figure 1A**). TRB inhibited early-stage biofilms of *T. mentagrophytes* ATCC 11481, *T. rubrum* ATCC MYA-4438, and *T. rubrum* ATCC 28289 from concentrations of 0.06, 1, and 32 mg/L, respectively (p < 0.001) (**Figure 1B**). However, FLZ only inhibited the biofilms formed by the *T. mentagrophytes* ATCC 11481 from 32 mg/L (p < 0.001) (**Figure 1C**).

**Figure 2:**
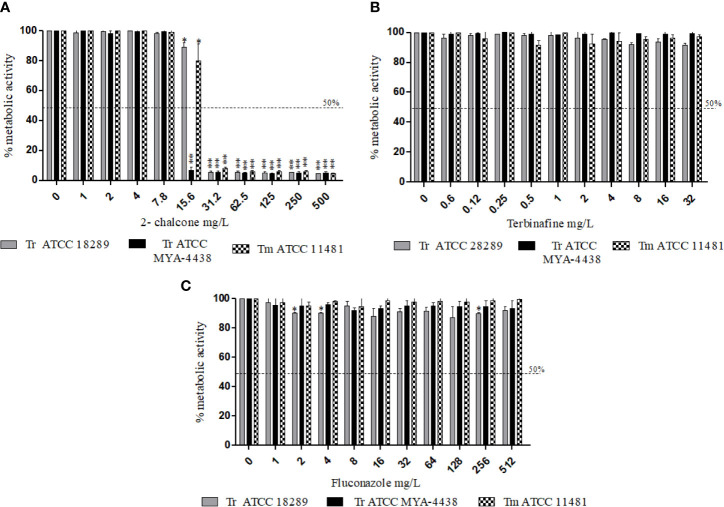
Effect of 2-chalcone **(A)**, terbinafine **(B)**, and fluconazole **(C)** on mature biofilms of *T. rubrum* ATCC 28189, *T. rubrum* ATCC MYA-4438, and *T. mentagrophytes* ATCC 11481 measured by the XTT reduction assay. Only 2-chalcone showed action against mature biofilms from a concentration of 31.2 mg/L. On the contrary, biofilms formed by all tested strains were resistant to the drugs terbinafine and fluconazole even at the highest concentrations (*p < 0.05; **p < 0.001). Tr- *T. rubrum*, Tm- *T. mentagrophytes*.

In mature biofilms, 2-chalcone showed potent anti-biofilm activity, and inhibited the metabolic activity by about 90% in all fungi tested from the concentration of 31.25 mg/L (p < 0.001) (**Figure 2A**). In contrast, TRB and FLZ did not show anti-biofilm activity at all the different concentrations tested (**Figures 2B, C**).

### Effect of 2-Chalcone and Antifungal Drugs on Planktonic Cells at a Concentration of 10^6^ cells/mL

**Figure 3:**
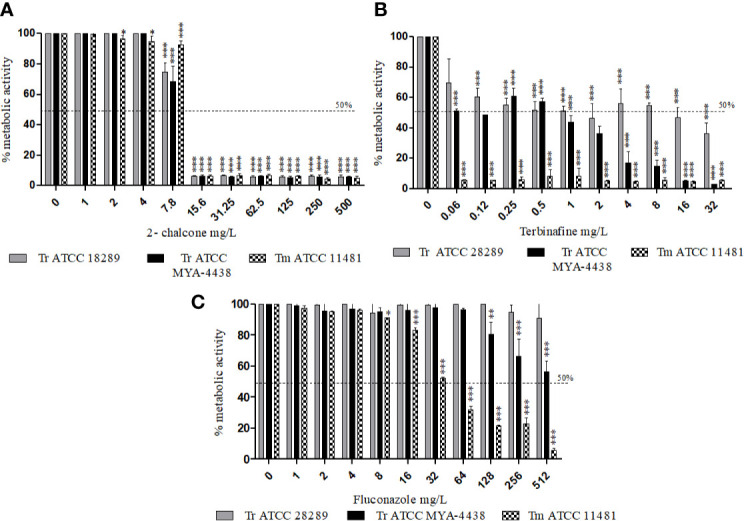
Effect of 2-chalcone **(A)**, terbinafine **(B)**, and fluconazole **(C)** on planktonic cells of *T. rubrum* ATCC 28189, *T. rubrum* ATCC MYA-4438, and *T. mentagrophytes* ATCC 11481 measured using the XTT reduction assay. The compounds 2-chalcone and terbinafine were more potent against planktonic cells than fluconazole. The compounds 2-chalcone, terbinafine, and fluconazole inhibited the cellular metabolic activity in the planktonic form at the concentration used for biofilm formation (10^6^ cells/mL) of all strains tested, from the concentration of 15.6, 0.06, and 512 mg/L respectively. (*p < 0.05; **p < 0.01; ***p < 0.001). Tr, *T. rubrum*, Tm- *T. mentagrophytes*.

The effect of 2-chalcone and of the drugs TRB and FLZ on planktonic cells was verified at the same concentration used for biofilm formation (1 × 10^6^ cells/mL) by measuring the metabolic activity of the cells. The results showed that 2-chalcone could reduce the metabolic activity of *T. rubrum* ATCC 28189 from the concentration of 7.8 mg/L (p < 0.001) of *T. rubrum* ATCC MYA-4438 and *T. mentagrophytes* ATCC 1148 from 15.6 mg/L (p < 0.001) (**Figure 3A**). TRB was potent in *T. mentagrophytes* ATCC 1148 with a reduction in metabolic activity from the concentration of 0.008 mg/L, and in *T. rubrum* ATCC MYA-4438 and *T. rubrum* ATCC 28189 from 0.03 mg/L and 0.06 mg/L, respectively (p < 0.001) (**Figure 3B**). Besides, FLZ was less potent than TRB, with reduced metabolic activity from the concentration of 64, 256, and 512 mg/L in *T. mentagrophytes* ATCC 1148, *T. rubrum* ATCC MYA-4438, and *T. rubrum* ATCC 28189, respectively (p < 0.001) (**Figure 3C**).

### Effect of Photodynamic Therapy on Planktonic Cells and on Early-Stage and Mature Biofilms

**Figure 4:**
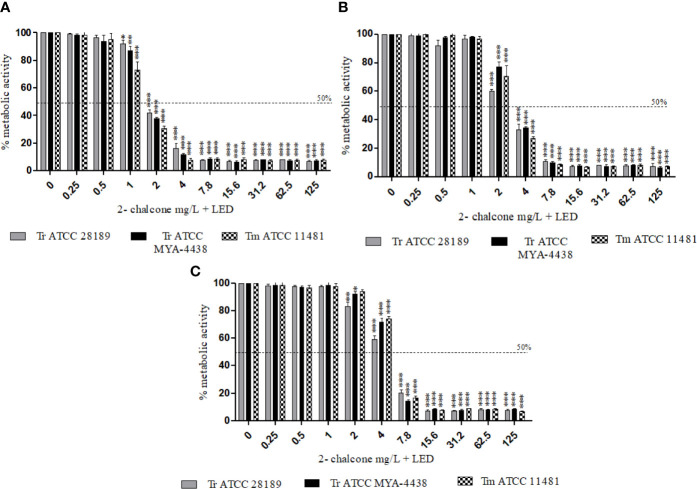
Effect of 2-chalcone-mediated PDT using LED irradiation at a dose of 150 J/cm^2^ in planktonic cells (10^6^ cell/mL) **(A)**, early-stage **(B)**, and mature biofilms **(C)** of *T. rubrum* ATCC 28189, *T. mentagrophytes* ATCC 11481, and *T. rubrum* ATCC MYA-4438, measured by the XTT reduction assay. Potentiation of 2-chalcone was shown when it was mediated PDT resulting in the inhibition of planktonic forms and biofilms (early-stage and mature) from 2, 4, and 7.8 mg/L. (*p < 0.05; **p < 0.01; ***p < 0.001).

The photodynamic therapy assay was applied using 2-chalcone as a photosensitizer against planktonic cells (10^6^ cells/mL) and against early-stage and mature biofilms of the strains *T. rubrum* ATCC 28189, *T. mentagrophytes* ATCC 11481, and *T. rubrum* ATCC MYA-4438. The use of 2-chalcone as a photosensitizer for PDT was found to be effective against dermatophytes (**Figure 4**). The metabolic activities of planktonic cells of all tested species were inhibited from the concentration of 2 mg/L (**Figure 4A**), corresponding to a four-times reduction in concentration compared to its effect in the dark. The same reduction was observed in early-stage biofilms with inhibition from 4 mg/L (**Figure 4B**) and in mature biofilms with inhibition from 7.8 mg/L (**Figure 4C**).

### Scanning Electron Microscopy of Biofilms Treated With 2-Chalcone in the Dark and Mediated PDT

**Figure 5:**
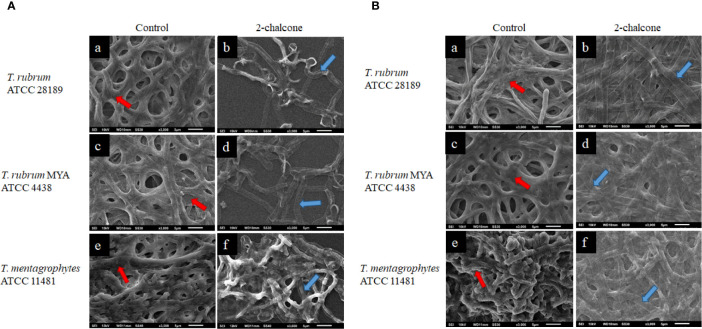
Scanning electron microscopy (SEM) images of early stage **(A)** and mature biofilms **(B)** of *T. rubrum* ATCC 28189, *T. rubrum* ATCC MYA-4438, and *T. mentagrophytes* ATCC 11481 untreated (a, c, e) and treated with 2-chalcone in the dark (b, d, f). The images of untreated biofilms show a robust biofilm, formed with the entanglement of integral hyphae and covered with a polymeric extracellular matrix (red arrows). Biofilms treated in the early-stage with 2-chalcone present a low density showing the action of 2-chalcone in inhibiting their maturation. In mature biofilms, as in the early-stage biofilms, 2-chalcone promoted total hyphal collapse (blue arrows).

SEM was used to evaluate the damage to *in vitro* early-stage and mature biofilms treated with 2-chalcone. For this analysis, the concentrations of 2-chalcone (in the dark and irradiated) determined in the susceptibility test were used. The topographies of biofilms treated with 2-chalcone confirmed the findings of the XTT reduction assay and showed a total collapse in the hyphal cell wall probably due to leakage of cytoplasmic content. These damages were observed in both early-stage biofilms (**Figure 5A**) and mature biofilms (**Figure 5B**). Further, evident inhibition of biofilm maturation and decreased presence of polysaccharide material was observed in the initial biofilms. On the contrary, untreated biofilms showed a dense network of interconnected hyphae embedded in an extracellular matrix [**Figures 5A** (e) **B** (a, c, e)].

**Figure 6:**
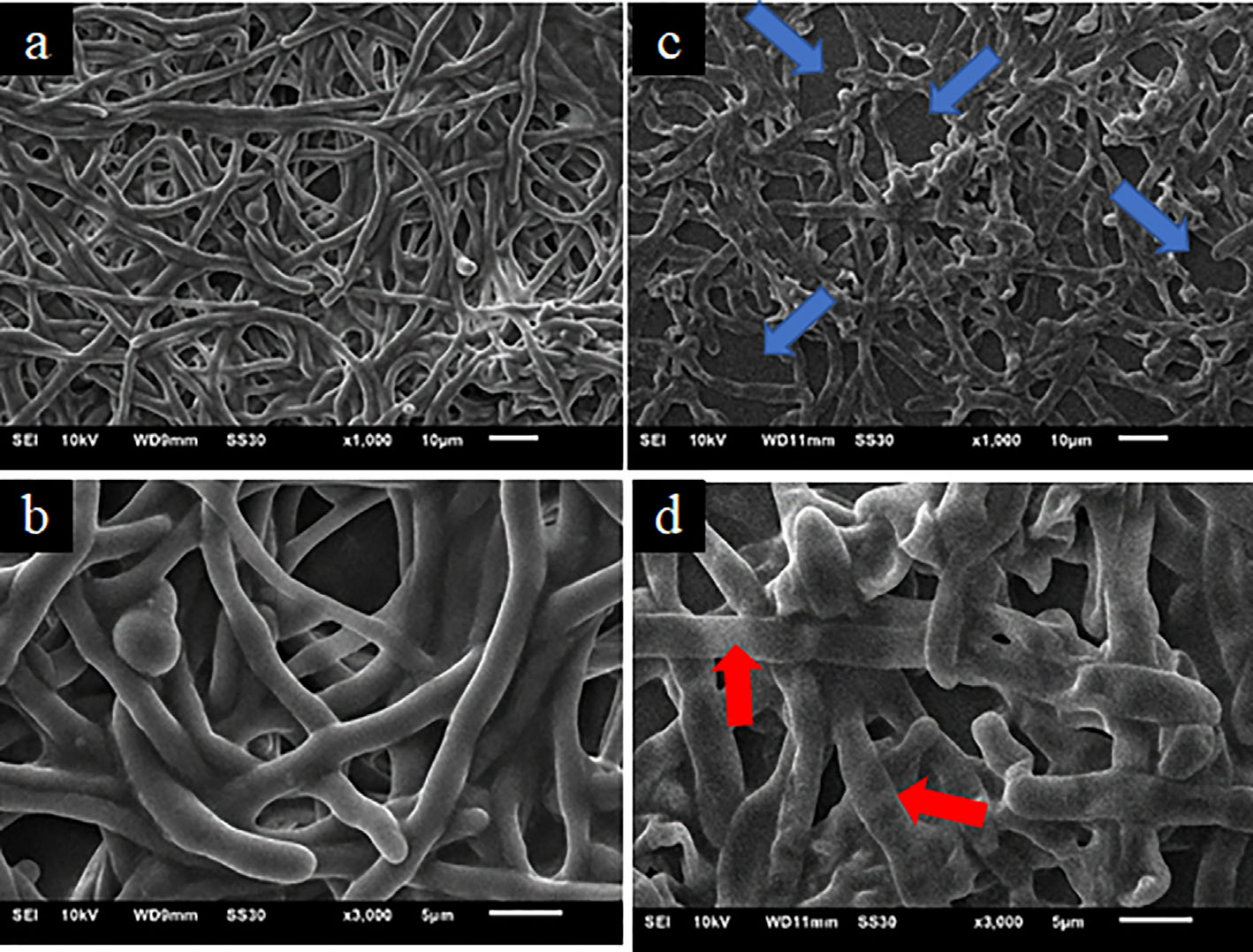
Scanning electron microscopy images of mature *T. rubrum* ATCC 28189 biofilms treated with 2-chalcone combined with blue LED at a dose of 150 J/cm^2^ **(C, D)** and irradiated only at a dose of 150 J/cm^2^ without the photosensitizer **(A, B)**. The blue arrows indicate empty spaces within the biofilm showing that the biofilm had become less dense. The red arrows show collapsed hyphae.

Photomicrographs of *T. rubrum* ATCC 28189 biofilms treated with 2-chalcone-mediated PDT (**Figure 6**) also confirmed the XTT assay findings. However, the biofilms were less dense, and the collapse of the hyphae cell walls was less prominent (**Figures 6C, D**). Biofilms treated at a dose of 150 J.cm^-2^ without the photosensitizer (**Figures 6A, B**) showed similar results to those without treatment in the dark, also corroborating the XTT assay results.

### Cytotoxicity Assay With 2-Chalcone in HaCat

**Figure 7:**
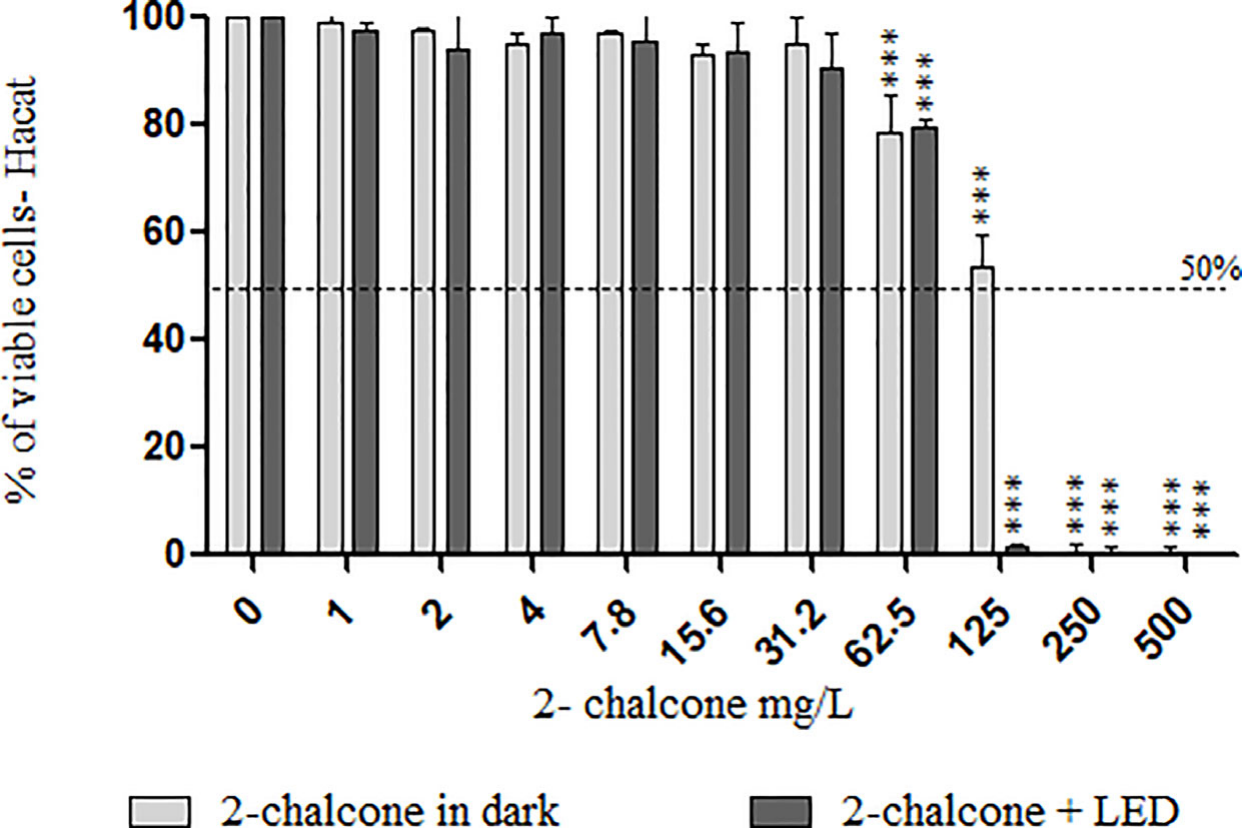
Viability of HaCat cells measured using the resazurin reduction method after contact with different concentrations of 2-chalcone in the dark and 2-chalcone photoexcited with blue LED at a dose of 150 J/cm^2^. The compound 2-chalcone decreased viability in PDT reducing the cell viability to 80% at a concentration of 62.5 mg/L when compared to the control without treatment. On the contrary, cells treated with 2-chalcone in the dark had viability greater than 50%, even at concentrations of 125 mg/L (***p < 0.001).

Treatment of keratinocytes with 2-chalcone in the dark, reduced viability by almost 50% at 125 mg/L compared to the control without treatment (**Figure 7**). Cells treated with 2-chalcone-mediated PDT showed high toxicity with only 3% viability at the same concentration. After calculating the SI, 2-chalcone-mediated PDT presented better values in planktonic cells (38.72) than in the mature biofilm (9.92) when compared to the 2-chalcone treatment in the dark (**Table 2**). Photoexcitation of the compound using a light source is promising as photosensitization resulted in significant potentiation and decreased cell toxicity.

**Table 2:** Values of IC_50_ and selectivity index (SI) in HaCat cell monolayers treated with 2-chalcone in the dark and with photosensitization.

Condition	IC_50_	SI (IC_50_/MIC) planctonic 10^6^ cel/mL			SI (IC_50_/MIC) mature biofilm		
		Tr ATCC 28189	Tr ATCC MYA4438	Tm ATCC 11481	Tr ATCC 28189	Tr ATCC MYA4438	Tm ATCC 11481
2-chalcone in dark	130.7	16.75	8.38	8.38	4.18	8.36	4.18
2-chalcone + LED (150 J/cm^2^)	77.44	38.72	38.72	38.72	9.92	9.92	9.92

IC_50_, concentration that promotes 50% inhibition of cellular activity. Tr, T. rubrum; Tm, T. mentagrophytes.

### Determination of the Mechanisms of Action

#### Verification of Damage to the Cell Membrane and Wall

**Figure 8:**
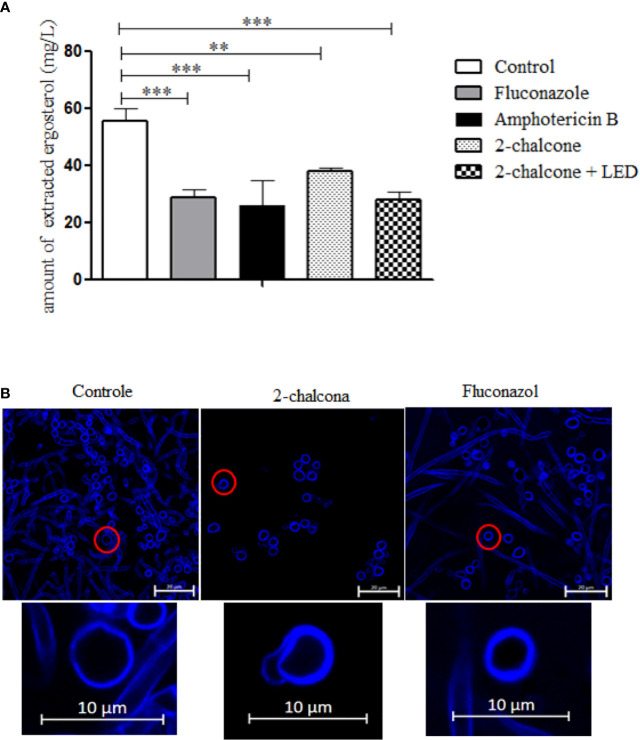
The amount of extracted ergosterol **(A)** and confocal laser scanning microscopy images (CLSM) **(B)** of *T. rubrum* ATCC 28189 treated with 2-chalcone, fluconazole, and amphotericin B. The ergosterol quantification graph shows that treatments with 2-chalcone in the dark and 2-chalcone-mediated PDT reduced the amount of sterols extracted, suggesting that this molecule may have an action on ergosterol or its synthesis chain. Amphotericin B and fluconazole also reduced the amount of steroids extracted, proving their direct action and on the synthesis chain, respectively. In CLSM images, the cell wall is stained with calcofluor white. Cells treated with 2-chalcone showed changes in the cell wall with compromised structure. However, cells treated with fluconazole showed cell wall integrity. **p < 0.01; ***p < 0.001.

The results of ergosterol quantification in the fungal membrane (**Figure 8A**) showed that 2-chalcone in the dark, as well as 2-chalcone-mediated PDT, reduced the amount of total sterols extracted compared to the control (p < 0.01). These findings revealed that ergosterol inhibition might be a mechanism of action for 2-chalcone. FLZ and AMB also reduced the number of sterols extracted from the membrane (p < 0.001).

Confocal microscopy images (**Figure 8B**) of cells treated with 2-chalcone showed discontinuous staining of the cell wall and structural compromise compared to the control indicating that 2-chalcone can damage cell wall chitin and/or cellulose. Cells treated with FLZ showed no change in the cell walls of the conidia and hyphae.

#### Quantification of ROS and Apoptosis/Necrosis

**Figure 9:**
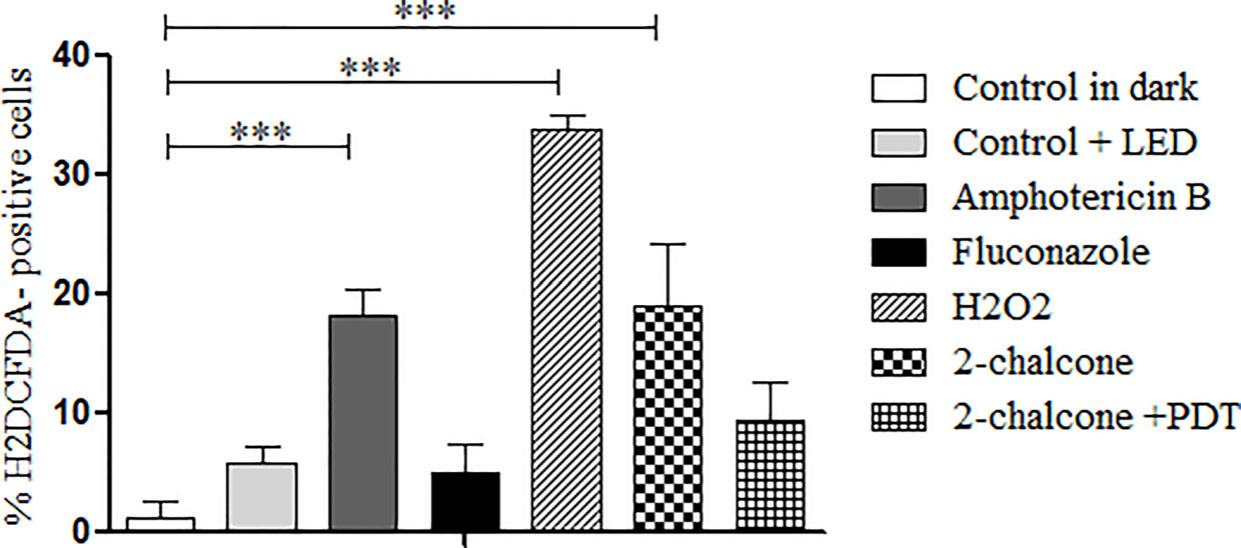
Measurement of ROS production after treatment of *T. rubrum* ATCC 28189 with 2-chalcone in the dark and mediated PDT (2-chalcone + LED). The compounds amphotericin B, hydrogen peroxide, and 2-chalcone in the dark induced ROS formation when compared to the control without treatment in the dark. Treatment with 2-chalcone-mediated PDT did not induce ROS formation when compared to the control + LED. ***p <0.001.

In the dark, 2-chalcone induced ROS generation when compared to the control (p < 0.001) (**Figure 9**); however, 2-chalcone-mediated PDT did not produce ROS. AMB and H_2_O_2_ also induced ROS formation when compared to the untreated control (p < 0.001). In contrast, FLZ did not induce ROS formation (**Figure 9**).

**Figure 10:**
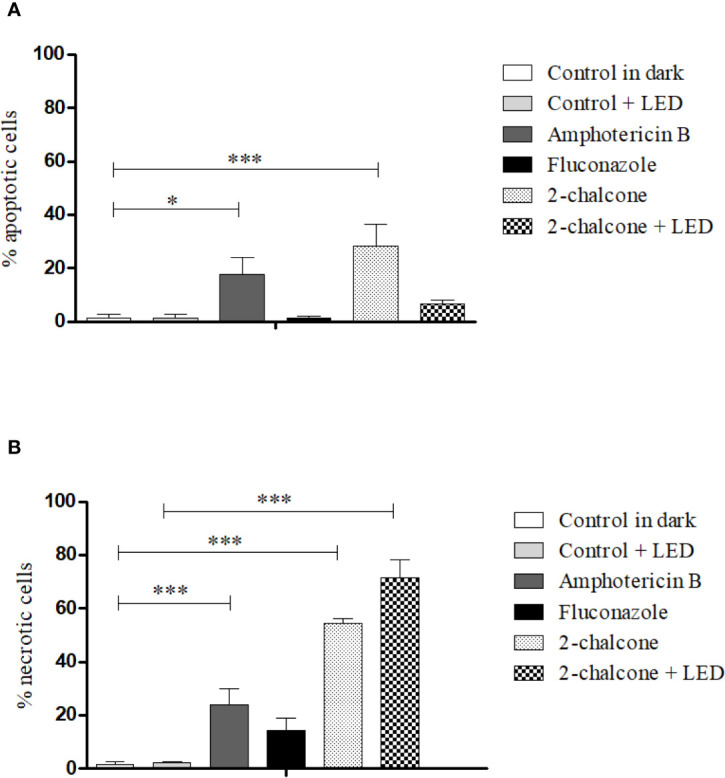
Mechanism of death due to apoptosis **(A)** and necrosis **(B)** induced after treatment of *T. rubrum* ATCC 28189 with 2-chalcone in the dark and with 2-chalcone mediated PDT (2-chalcone +LED), compared with the untreated control. Further, 2-chalcone in the dark and amphotericin B induced cell death by both apoptosis and necrosis, whereas 2-chalcone +LED only induced death by necrosis. *p < 0.05; ***p < 0.001.

Cells treated with 2-chalcone in the dark presented high necrosis levels (53–56.5%) compared to death by apoptosis (18.4–32.3%) (p < 0.001) (**Figure 10A**). When 2-chalcone was excited by a light source, almost all cell death was found to be due to necrosis (**Figure 10B**). AMB caused death through both mechanisms, apoptosis (p < 0.05) and necrosis (p < 0.001). In contrast, most FLZ-treated cells remained alive because the *T. rubrum* ATCC 28189 strain was resistant to FLZ as shown in the susceptibility assay. However, a tendency of cellular death mainly by necrosis, was observed (**Figure 10**).

## Discussion

Extensive research has been conducted on new anti-dermatophyte drugs (Gupta; Foley; Versteeg, 2017b; Gnat; Lagowski; Nowakiewicz, 2020; Iwanaga et al., 2020) as microorganisms are increasingly developing resistance to conventional drugs with the hypothetical formation of biofilms in onychomycosis and high rates of recurrence and reinfection (Gupta; Daigle; Carviel, 2016; Gupta; Foley, 2019). Further, the increasing human infections by zoophilic species, mainly in *tinea capitis* and *tinea unguium*, are generally more challenging to treat and require systemic treatment (Gnat; Lagowski; Nowakiewicz, 2020).

The present work showed the anti-dermatophyte and anti-biofilm action of a compound derived from chalcone, a molecule of natural origin that is abundant in fruits and vegetables and has relatively simple laboratory synthesis. The compound shows enhanced action mediated PDT, with a better SI in human keratinocytes in the context of its effect in the dark. Tests of susceptibility and MFC determination showed the potent action of 2-chalcone with a MIC of 7.8 mg/L in all tested strains. Although the action of chalcones varies widely with their structural replacement pattern, natural and synthetic chalcones have already shown antifungal activity against *Candida* spp (Tavares et al., 2011), *Cryptococcus gattii* (Palanco et al., 2017), *Paracoccidioides brasiliensis* (Medina-Alarcón et al., 2020), *H. capsulatum* (Melo et al., 2017), and against dermatophytes (López et al., 2001; Gupta; Jain, 2015), proving to be potential candidates as future antifungal drugs. The MFC is defined as the lowest concentration of the drug necessary to inhibit 99.9% of fungal growth (Gil-Alonso et al., 2019). Hazen (1998) considered an antifungal agent as fungicidal if the MIC and MFC relationship is not greater than four times. In all tested strains, 2-chalcone presented a potent fungicidal profile, with the relation between MIC and MFC equal to 2 times. The TRB and FLZ susceptibility of all strains corroborated with previous studies conducted by Costa-Orlandi et al. (2020).

Further, 2-chalcone also showed potent action against early-stage and mature biofilms, as well as the planktonic form with 10^6^ cells/mL with variations of only two dilutions. Costa-Orlandi et al. (2020) and Singulani et al. (2018) considered insignificant variations in dilutions up to two dilutions. These results are encouraging as no effect was observed with the drugs TRB and FLZ, mainly when used to treat mature biofilms. Some studies conducted by our group have already demonstrated the anti-biofilm activity of chalcones against fungal species such as *C. gattii* (Palanco et al., 2017) and *H. capsulatum* (Melo et al., 2017). Studies have been carried out to assess the anti-biofilm action of conventional drugs for treating dermatophytoses such as TRB, FLZ (Costa-Orlandi et al., 2020), itraconazole, voriconazole, griseofulvin (Brilhante et al., 2018), econazole (Toukabri et al., 2018), and formulations of piroctone-based shampoos (Santos; Dias-Souza, 2017). However, in most cases, 50 times the MIC is required to observe any biofilm inhibition (Brilhante et al., 2018). Planktonic forms (10^6^ cells/mL) are more sensitive to drugs when compared to biofilms, confirming that these communities show increased resistance to conventional antifungals. The strain *T. rubrum* ATCC 28189 demonstrated resistance to FLZ even in the planktonic form as described previously (Costa-Orlandi et al., 2020).

PDT is a relatively affordable treatment when used as an inexpensive photosensitizer and can be implemented in hospitals without incurring high costs (Bacellar et al., 2015). Some studies have shown the fungicidal effect of photodynamic therapy against *T. rubrum* and *T. mentagrophytes* using CO_2_ (De Oliveira et al., 2015) and toluidine (Baltazar et al., 2013), as well as with hypericin, hypocrillin, and curcumin as photosensitizers against *Candida* spp. (Davies et al., 2016; Yang et al., 2019). Cyclic chalcones have significant characteristics for effects in PDT (Melo et al., 2017). However, the photosensitizing properties of chalcones can be easily lost by the modification of their structures. For instance, Zhuang et al. (2018) reported that the introduction of a methyl group at the α position of the unsaturated ketone resulted in the loss of fluorescence. The usage of 2-chalcone as a photosensitizer in PDT showed that the compound did not lose its photosensitizing properties. Further, its effect was enhanced compared to that in the dark with a reduction in the MIC to a fourth part against planktonic cells, early-stage and mature biofilms.

Photosensitization with 2-chalcone showed increased toxicity in HaCat cells, with an IC_50_ reduction by almost by half. Similar results have been reported by Melo et al. (2017) when irradiated chalcone derivatives were co-incubated with NOK, HepG2, and HaCat cell lines at a dose of 12 and 42 J.cm^-2^. The researchers also found that the dose of light was directly proportional to the increase in cell toxicity. In contrast, the SI in the treatment of 2-chalcone-mediated PDT was higher than that in the dark treatment. The SI is the ratio of the IC_50_ and MIC (Scorzoni et al., 2016). This index indicates a compound’s selectivity between a fungal and host cell, to evaluate the relationship between safety and potency (Bagla et al., 2014; Scorzoni et al., 2016). SI values higher than ten are considered more specific (Ochoa-Pacheco et al., 2017; Singulani et al., 2019). Treatment of 2-chalcone-mediated PDT proved to be safer, with SI values ranging from 9.92 to 38.72 in mature biofilms and planktonic forms, respectively. These results are a consequence of the decreased MIC values in planktonic cells and biofilms, and the reduced toxicity in HaCat cells.

The antifungal properties of chalcones depend on their structural replacement pattern, as well as on the fungal genotype and cell density (López et al., 2001; Illicachi et al., 2017). The action on the biosynthesis of β- (1,3) glucan and chitin of the fungal cell wall (Gupta; Jain, 2015; Illicachi et al., 2017) has already been demonstrated, also the inhibition of the glutathione-S-transferase (GST) family, that are enzymes involved in drug resistance (Illicachi et al., 2017). Due to the diversity of mechanisms underlying the action of chalcones, our work verified the integrity of structures such as cell walls and cell membranes as well as the functional imbalance including oxidative stress induction and cell death mechanism in the treated samples. Treatment with 2-chalcone in the dark and 2-chalcone-mediated PDT reduced membrane ergosterol contents. The action of chalcones on this molecule has not been reported so far. Lack of ergosterol alters membrane fluidity, causing an increase in permeability and consequent osmotic imbalance (Ouf et al., 2013). Cells treated with AMB and FLZ also showed lower amounts of ergosterol because these drugs act by binding directly to membrane ergosterol and inhibiting its synthesis, respectively (Singulani et al., 2019). Further, our results showed deformation of the cell wall structure when stained with calcofluor white, a non-specific fluorophore that binds cellulose and chitin mainly in beta 1-3 and beta 1-4 polysaccharides, and emit fluorescence when excited (Harrington; Hageage, 2003). The action of chalcones on the fungal cell wall has been reported previously (Gupta; Jain, 2015; Illicachi et al., 2017). Reduced ergosterol and impaired cell wall structure confirm the findings in the SEM images showing that the hyphae were fully collapsed with a “pressed cell” appearance.

Treatment with 2-chalcone in the dark induced significant ROS generation. Drugs such as amphotericin B have ROS induction as a secondary mechanism of action, in addition to the main mechanism *via* ergosterol (Singulani et al., 2019). ROS generation depends on the drug’s ability to reach the intracellular region (Yoo; Ha, 2012). Possibly, a part of the compound that reached the cytoplasm was responsible for ROS generation. However, a significant part acts on the cell wall and membrane. Treatment with AMB as well as H_2_O_2_ induced ROS production. Further, AMB caused death by apoptosis as well as necrosis. Similar results were reported by Singulani et al. (2019)  in *C. neoformans*. The presence of oxygen in the PDT takes the photosensitizer to an excited state with high reactivity, facilitating interactions with the surrounding molecules (Kwiathowski et al., 2018; Donohoe et al., 2019). These interactions can be type I or type II. Type I interactions result in free radicals whereas type II interactions induce formation of ROS such as singlet oxygen (^1^O_2_), superoxide, hydrogen peroxide, and hydroxyl radical (Shibu et al., 2013; Zhang et al., 2018a). Our results showed that 2-chalcone mediated PDT promoted low ROS induction. Considering these facts, we speculate that the photosensitized 2-chalcone probably induces free radicals, through type I reactions.

Apoptosis and necrosis are the main mechanisms of cell death in cytotoxic responses to PDT. These depend on the photosensitizer nature, light dose, and cell type (Yoo; Ha, 2012). Treatment with 2-chalcone-mediated PDT almost entirely induced fungal death by necrosis. Necrosis is usually associated with a high concentration of photosensitizer and/or light dose, severe cell damage, and photosensitizers with tropism to the cell membrane (Yoo; Ha, 2012). A relatively high dose (150 J.cm^-2^) was used in our assays, and the results showed that 2-chalcone has a tropism for the fungal membrane and cell wall. Induction of apoptotic death by the compound in the dark is probably a consequence of ROS generation. However, most cells died from necrosis, which may be a result of other cell targets such as the cell wall and the fungal membrane ergosterol.

## Conclusion

Our results showed that 2-chalcone is a molecule with anti-dermatophyte and anti-biofilm properties. When mediated PDT, its effect is enhanced, causing low toxicity to human skin keratinocytes and high SI value. Further, the compound targets specific fungal structures and promotes ROS generation, resulting in cell death from apoptosis and necrosis. Overall, this study contributes significantly to the discovery of new compounds with anti-biofilm activity, and other studies are being conducted to prove these findings both *ex vivo* and *in vivo*.

## Data Availability Statement

The original contributions presented in the study are included in the article/supplementary material. Further inquiries can be directed to the corresponding author.

## Author Contributions 

NB, CC-O, CF, and MM-G conceived and designed the study. NB, CC-O, CV, and JB performed all the experiments. NB, CV, and CC-O analyzed the data and wrote the manuscript. LA and LR synthesized 2-chalcone. All authors contributed to the article and approved the submitted version.

## Funding

This work was supported by Fundação de Amparo à Pesquisa do Estado de São Paulo-FAPESP [2019/22188-8 (NB), 2018/02785-9 (MM-G), 2017/18388-6 (CC-O), 2020/15586-4 (CV)], Programa de Apoio ao Desenvolvimento Científico (PADC) da Faculdade de Ciências Farmacêuticas da UNESP, Coordenação de Aperfeiçoamento de Pessoal de Nível Superior (CAPES) [Finance code 001; 88887.500765/2020-00 (CV)], Conselho Nacional de Desenvolvimento Científico e Tecnológico (CNPq) [142049/2019- 0 (NB), 105072/2018-4 (JB), 134559/2018-5 (CV)], and Instituto de Bolsa de Estudos (IBE) - Moçambique [IBE150/2017 (NB)].

## Conflict of Interest

The authors declare that the research was conducted in the absence of any commercial or financial relationships that could be construed as a potential conflict of interest.
